# Rabies surveillance in dogs in Lao PDR from 2010-2016

**DOI:** 10.1371/journal.pntd.0005609

**Published:** 2017-06-01

**Authors:** Bounlom Douangngeun, Watthana Theppangna, Phouvong Phommachanh, Keo Chomdara, Sithong Phiphakhavong, Syseng Khounsy, Mavuto Mukaka, David A. B. Dance, Stuart D. Blacksell

**Affiliations:** 1 National Animal Health Laboratory, Department of Livestock and Fisheries, Ministry of Agriculture, Ban Khunta, Vientiane, Lao People’s Democratic Republic; 2 Mahidol-Oxford Tropical Medicine Research Unit, Faculty of Tropical Medicine, Mahidol University, Bangkok, Thailand; 3 Centre for Tropical Medicine & Global Health, Nuffield Department of Medicine, University of Oxford, Old Road Campus, Oxford, United Kingdom; 4 Lao-Oxford-Mahosot Hospital-Wellcome Trust Research Unit (LOMWRU), Mahosot Hospital, Vientiane, Lao People’s Democratic Republic; 5 Faculty of Infectious and Tropical Diseases, London School of Hygiene and Tropical Medicine, London, United Kingdom; Colorado State University, UNITED STATES

## Abstract

**Background:**

Rabies is a fatal viral disease that continues to threaten both human and animal health in endemic countries. The Lao People’s Democratic Republic (Lao PDR) is a rabies-endemic country in which dogs are the main reservoir and continue to present health risks for both human and animals throughout the country.

**Methods:**

Passive, laboratory–based rabies surveillance was performed for suspected cases of dog rabies in Vientiane Capital during 2010–2016 and eight additional provinces between 2015–2016 using the Direct Fluorescent Antibody Test (DFAT).

**Results:**

There were 284 rabies positive cases from 415 dog samples submitted for diagnosis. 257 cases were from Vientiane Capital (2010–2016) and the remaining 27 cases were submitted during 2015–2016 from Champassak (16 cases), Vientiane Province (4 cases), Xieng Kuang (3 cases), Luang Prabang (2 cases), Saravan (1 case), Saisomboun (1 case) and Bokeo (1 case). There was a significant increase in rabies cases during the dry season (p = 0.004) (November to April; i.e., <100mm of rainfall per month). No significant differences were noted between age, sex, locality of rabies cases.

**Conclusion:**

The use of laboratory-based rabies surveillance is a useful method of monitoring rabies in Lao PDR and should be expanded to other provincial centers, particularly where there are active rabies control programs.

## Author summary

Rabies is a viral disease that continues to threaten both human and animal health in endemic countries and almost always results in death of the infected individual. In most areas of Southeast Asia, rabies continues to be a major zoonosis with the main source of human cases being dog bites. In this study, we examined the brains of 415 rabies-suspected dogs from Vientiane Capital and some other provinces of Lao PDR during 2010–2016. Overall, rabies was confirmed in 284 cases. We found that there was a significant increase in rabies cases during the dry season (November to April) when monthly rainfall was less than <100mm. The laboratory-based surveillance of rabies in the domestic animal population is an important component of a rabies control program, however it should be used in conjunction with an effective vaccine program.

## Introduction

Rabies is an acute viral encephalomyelitis caused by the rabies virus (genus Lyssavirus; family *Rhabdoviridae*) which can affect all warm-blooded animals including humans. Humans and animals may be infected with rabies virus via saliva through a bite or scratch [1] or butchering [2] of a rabid animal, and if left untreated it almost invariably leads to a fatal outcome. The World Health Organization estimates approximately 30,000 human deaths per year due to canine rabies in Asia out of a total 61,000 deaths per year due to rabies worldwide [3].

The Lao People’s Democratic Republic (Lao PDR) is a rabies-endemic country in which dogs are the main reservoir and continue to present health risks for both human and animals throughout the country [4, 5]. Historical reports of rabies cases in Lao PDR have been recorded in humans since 1963 [6] and rabies cases in dogs have been reported since the late 1960s [7]. It has been estimated that an average of 8,528 dog bites are reported annually in Lao PDR [8] underlining the importance of rabies surveillance and control. More recently, studies have identified the increasing importance of dog rabies in Lao PDR [4], Cambodia [9], and southern China [10], examining diagnostic issues, case numbers, epidemiology and genetic relatedness of contemporary regional strains.

Here we present results that 1) describe the number of dog rabies cases confirmed in Lao PDR from 2010–2016 using laboratory-based surveillance in Vientiane Capital, and including samples submitted from eight central, northern and southern provinces; 2) investigate aspects of dog rabies cases in Lao PDR including age, sex, location and season; 3) discuss opportunities for control of rabies in domestic animals using vaccination and identify constraints in the context of a low-resource environment.

## Materials and methods

### Data for laboratory-based surveillance of rabies

**Fig 1:**
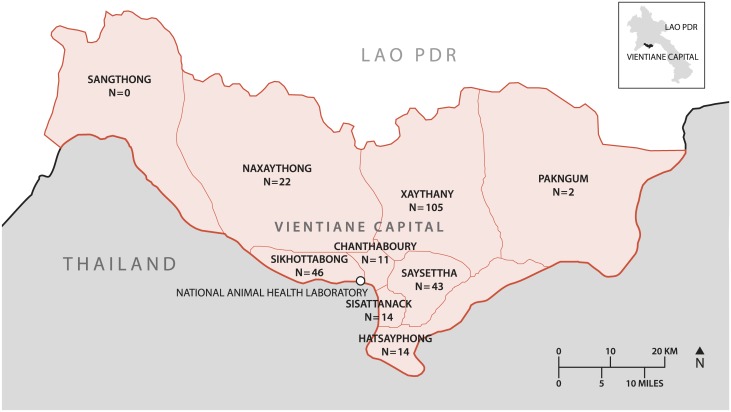
Map of Vientiane Capital indicating the number of rabies cases in each district in the period from 2010 to 2016.

Diagnostic testing for the presence of rabies virus was performed at the National Animal Health Laboratory (NAHL), Ban Khunta, Sikhottabong district, Vientiane Capital, Lao PDR (see Fig 1 for location).

**Fig 2:**
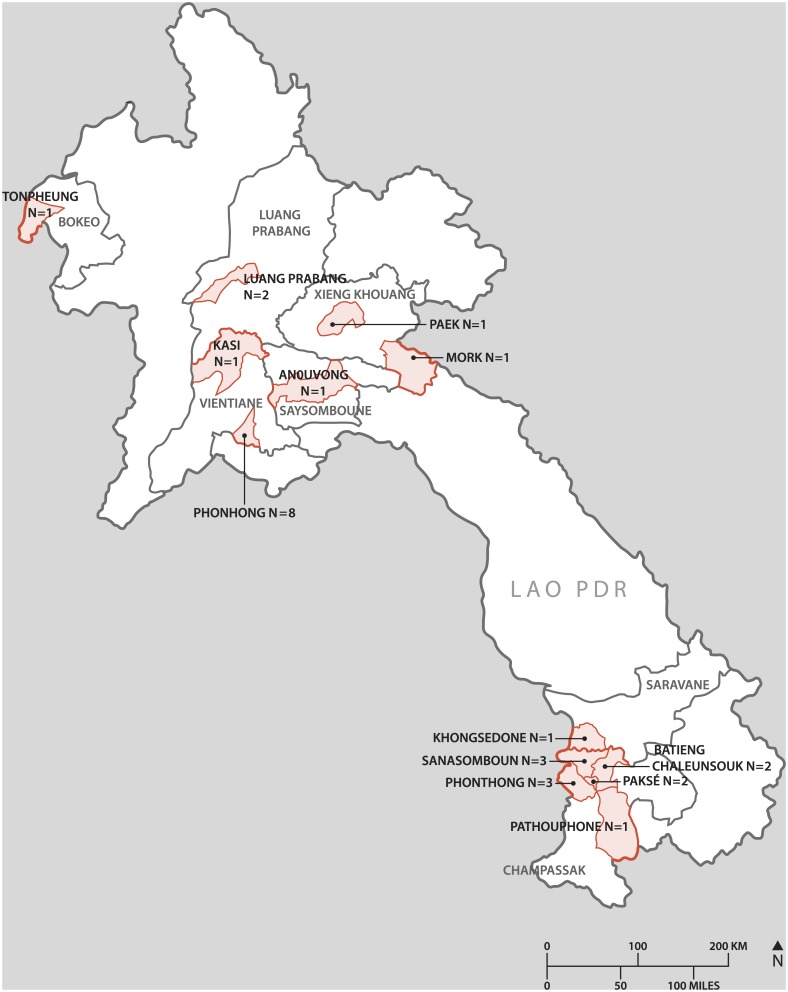
Map of Lao PDR indicating the number of rabies cases in each districts located outside of Vientiane Capital in the period from 2015 to 2016.

Rabies-suspected animals originated from the nine districts of Vientiane Capital (Fig 1) during 2010–2016. From 2015–2016, samples were also submitted from Champassak, Luang Prabang, Saisomboun, Vientiane Province, Xieng Kuang, Bokeo, Saravan and Sekong provinces (Fig 2).

Samples (carcass or head) presented for diagnosis came from animals that were strongly suspected of having rabies, exhibiting furious or comatose clinical signs, with the former often resulting in a human bite incident requiring rabies virus exclusion. In these circumstances, the bite-victim, and in many cases the animal owner, delivered the animals for urgent diagnostic testing. Rabies diagnostics on carcass material was performed following verbal agreement and consent of the animal owner and the bite victim so that compensation and treatment could be determined. Necropsy procedures were performed to allow sampling of the brain and collection of hippocampal tissue for diagnostic evaluation using the rabies virus direct fluorescent antibody test (DFAT) [11]. The DFAT requires a tissue impression or smear on a microscope slide stained with a FITC anti-rabies monoclonal antibody conjugate that is specific for rabies virus (Fujirebio Diagnostics Inc., USA) which is visualized by fluorescent microscopy.

### Data analysis

All analysis was performed using STATA 14.0 (StataCorp, College Station, TX USA). Descriptive statistics were used to characterise the number of rabies cases according to sex, age group, temporal and spatial distribution of rabies positive cases from 2010 to 2016. Significant differences (p<0.05) in rabies positive cases was determined using Pearson’s Chi-Square (Chi^2^) analysis. Age was compared using the groupings of <3, 3–12, >12–24, >24–36 and >36 months. Factors compared included the seasonal influence on rabies positive cases (wet season = >100mm of monthly rainfall i.e. May to October, compared to the dry season from November to April = <100mm of monthly rainfall [12]). Differences in the proportion of rabies positive samples from urban (Chanthabouly, Sisttanak, Sikhottabong and Saysettha) and rural (Naxaithong, Xaythany, Hatxaifong, Pakngum and Sangthong) districts of Vientiane Capital were also examined. Trends in the number of annual rabies cases and dog age were tested for significance (p<0.05) using the *ptrend* command which calculates a Chi^2^ statistic for trend.

### Historical data collection

Historical rabies case data from 1988–2011 was collected for comparative purposes. Data was derived from the same source being directly, or quoted as, provided by the Department of Livestock and Fisheries (DLF) of the Ministry of Agriculture, Lao PDR or by the Livestock and Veterinary Department (former title of DLF) from conference proceedings [13, 14], and a peer reviewed publication [4](Table 1). Data presented in Table 1 varies in terms of the number and provinces and species from where samples were submitted for diagnosis.

**Table 1:** Historical and contemporary reports of canine rabies confirmed cases in Lao PDR.

Year	No. samples tested	No. Provinces sampled	Species tested	No. rabies positive samples	% rabies positive	Diagnostic test	Reference
1988	90	1	Dog	83	92.2%	Not stated	[14]
1989	96	1	Dog	89	92.7%	Not stated	[14]
1990	107	1	Dog	97	90.1%	Not stated	[14]
1991	112	1	Dog	102	91.1%	Not stated	[14]
1992	144	1	Dog	136	94.4%	Not stated	[14]
1996	173	1	Dog	144	83%	Negri/mouse*	[13]
1997	219	1	Dog	103	47%	Negri/mouse	[13]
1998	200	1	Dog	96	48%	Negri/mouse	[13]
1999	200	1	Dog	70	35%	Negri/mouse	[13]
2000	165	1	Dog	50	30%	Negri/mouse	[13]
2004	163	7	Dog/Rat	66	40.5%	DFAT**	[4]
2005	167	5	Dog	84	50.3%	DFAT	[4]
2006	180	8	Dog/Rat	87	48.3%	DFAT	[4]
2007	152	6	Dog/cat	64	42.1%	DFAT	[4]
2008	199	7	Dog/Cat	103	51.7%	DFAT	[4]
2009	181	6	Dog/Cat/Monkey/Rabbit	109	60.2%	DFAT	[4]
2010	118/75***	5/1	Dog/Cat	72/53***	61.0%/70.7%	DFAT	[4]/This study
2011	101/63***	5/1	Dog	60/35***	59.4%/56.5%	DFAT	[4]/This study
2012	55	1	Dog	32	58.2%	DFAT	This study
2013	57	1	Dog	42	73.7%	DFAT	This study
2014	42	1	Dog	29	69.1%	DFAT	This study
2015	73	7	Dog	57	78.1%	DFAT	This study
2016	51	4	Dog	36	70.6%	DFAT	This study

* Negri/mouse—Observation of Negri bodies and mouse inoculation

**DFAT—Direct fluorescent antibody test

*** Data derived from the same source. % rabies positive difference due to variation in number of provinces and species sampled.

## Results

### Overall

During 2010–2016, 415 dogs or specimens from dogs were submitted to NAHL for rabies diagnosis. The overall number of rabies positive cases was 284 (68.4%).

### Annual results

**Fig 3:**
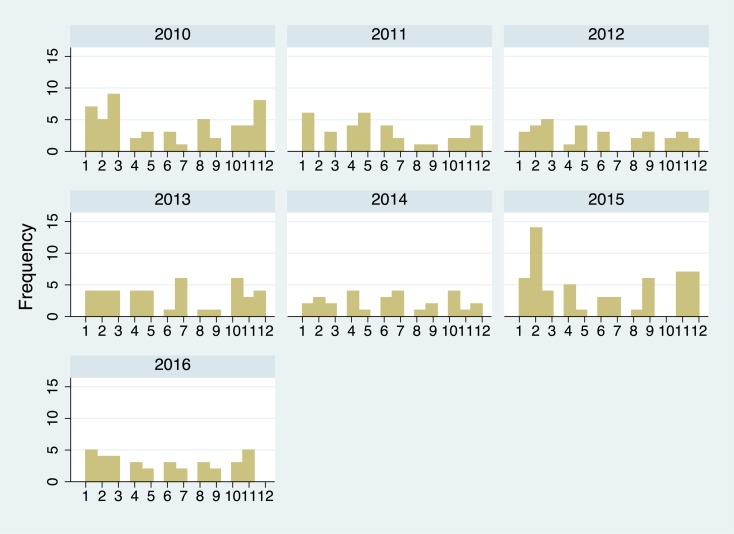
Frequency of rabies cases in Vientiane Capital province in each district in the period from 2010 to 2016.

The numbers of samples submitted for rabies testing annually and the numbers of confirmed rabies cases is presented in Table 1. The median number of samples submitted annually was 57 (IQR: 51–73), with a range from 42 (2014) to 75 (2010). The median annual number of confirmed rabies cases was 36 (IQR: 32–53), range 29 (2014) to 57 (2015), and the annual proportion of rabies positive samples ranged from 56.5% (2012) to 78.1% (2015). There was no significant difference in rabies positivity rates (Chi^2^ = 10.96; p = 0.009) with no significant trend for the seven-year sampling period (Chi^2^ = 0.497; p = 0.481). The numbers of confirmed rabies cases by month from 2010–2016 are presented in Fig 3.

### Geography

During the entire 2010–2016 sampling period, the preponderance of dog rabies cases came from Vientiane Capital (90.5%; 257/284) (Table 2) and of these majority were from Xanythany (37.0%; 105/284), Sikhottabong (16.2%; 46/284), Saysettha (15.1%; 43/28) and Naxaithong (7.8%; 22/284) districts. Fewer rabies cases were recorded from dogs in Hatxaifong (4.9%), Sisattanak (4.9%), Chantabouly (3.9%) and Pakngum (0.7%) districts, with no cases from Sangthong district (Table 2 and Fig 1). From 2015–2016, samples submitted from 8 provinces outside Vientiane Capital had at least one rabies case with the majority coming from the southern province of Champassak (6.6%; 16/284) and the centrally located Vientiane Province (1.4%; 4/284) (Table 2 and Fig 2).

**Table 2:** Total rabies-positive cases submitted to NAHL for 2010–2016 from the provinces and districts of Lao PDR.

Province	Rabies cases (%)	District	Rabies cases	Location
Vientiane Capital	257 (90.5%)	Chanthabouly	11	Urban
		Saysettha	43	Urban
		Sikhottabong	46	Urban
		Sisattanak	14	Urban
		Hatxaifong	14	Rural
		Naxaithong	22	Rural
		Pakngum	2	Rural
		Sangthong	0	Rural
		Xaythany	105	Rural
Champassak*	16 (5.6%)	Pakse	2	Urban
		Bachieng	2	Rural
		Phonthong	8	Rural
		Sanasomboun	3	Rural
		Pathoumphone	1	Rural
Luang Prabang*	2 (0.7%)	Luang Prabang	2	Urban
Saisomboun*	1 (0.35%)	Anouvong	1	Rural
Vientiane Province*	4 (1.4%)	Kasy	1	Rural
		Phonhong	3	Rural
Xieng Kuang*	2 (0.7%)	Mork	1	Rural
		Paek	1	Rural
Bokeo*	1 (0.35%)	Tonpheung	1	Rural
Saravan*	1 (0.35%)	Khongsedone	1	Rural
Sekong*	0	Lanam	0	Rural
Total	284			

*Samples only collected 2015–2016

The number of rural rabies cases was higher than that of urban districts (i.e., 166 *cf* 118) although this was not statistically significant (Chi^2^ = 0.044; p = 0.834). Focusing on rabies cases in Vientiane Capital, despite the larger number of samples from Xanythany, Sikhottabong, Saysettha and Naxaithong districts there was no statistical difference in the proportion of rabies cases between the districts (Chi^2^ = 12.4; p = 0.133) (Table 3). Fig 3 demonstrates annual frequency of rabies cases for the Vientiane Capital districts from 2010–2016.

**Table 3:** Descriptive features of animals submitted to NAHL 2010–2016.

Descriptive feature	Variable	*n* total samples (%)	*n* Rabies positive (%)	Chi^2^	Significance (*p*)
Sex*	Males	242 (59.9%)	172 (62.3%)	2.12	0.145
	Females	162 (40.1%)	104 (37.7%)		
Age	<3 months	40 (9.7%)	23 (57.5%)	4.32	0.363
	3–12 months	248 (60.2%)	167 (67.3%)		
	>12–24 months	57 (13.8%)	42 (73.7%)		
	>24–36 months	46 (11.2%)	35 (76.1%)		
	>36 months	21 (5.1%)	14 (66.7%)		
Location	Rural	244 (58.8%)	166 (68.0%)	0.044	0.833
	Urban	171 (41.2%)	118 (69.0%)		
Season	Wet**	182 (43.9%)	111 (61.0%)	8.32	0.004
	Dry	233 (56.1%)	173 (74.3%)		

* 11 animals not sexed

** >100mm of monthly rainfall

### Seasonality

**Fig 4:**
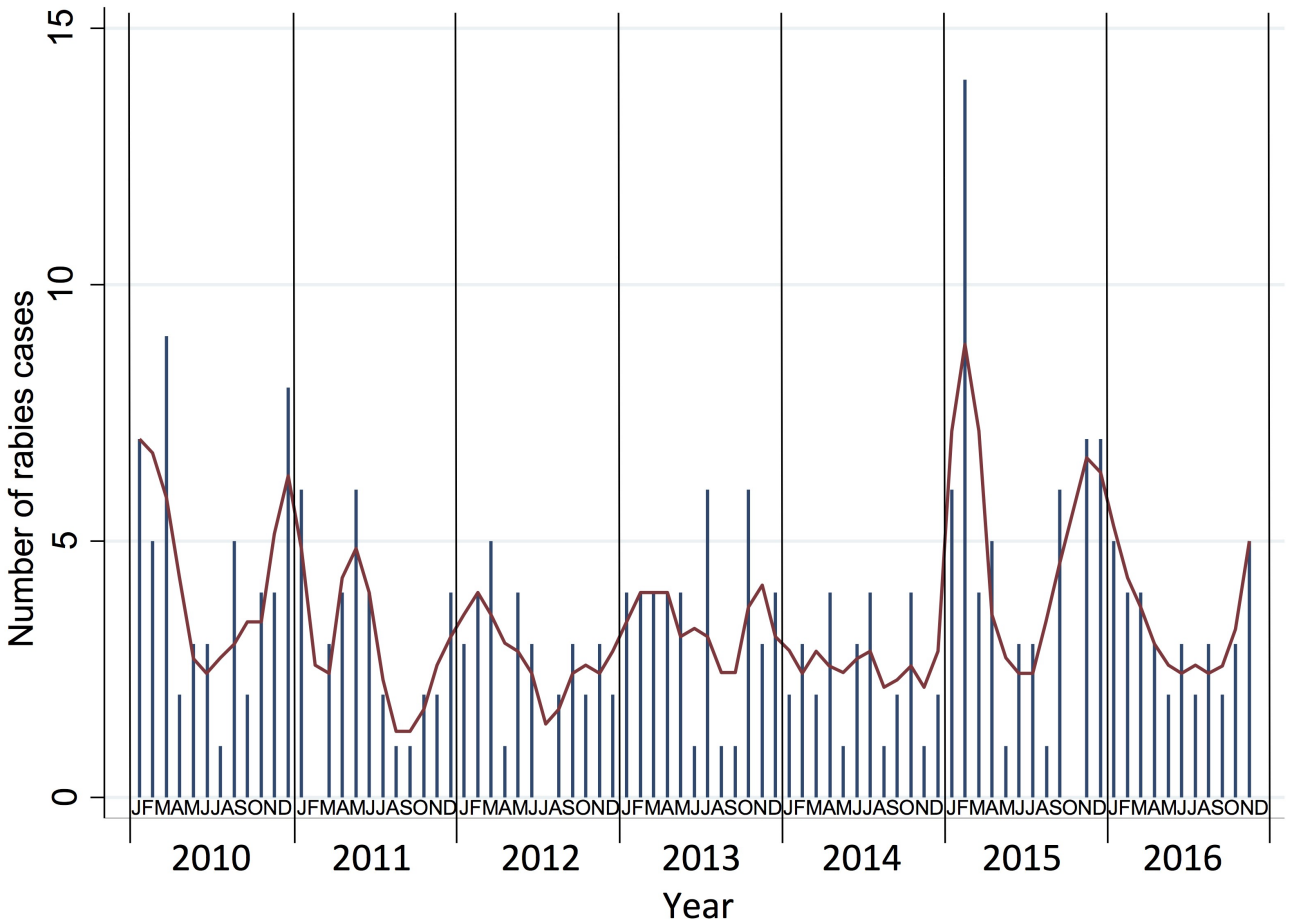
Annual monthly frequency of rabies cases for the period of 2010 to 2016 demonstrating the seasonality of rabies infections in Lao PDR. A running average of rabies cases is included as the red line.

There was a significant seasonal difference in the number of rabies cases (Chi^2^ = 8.32; p = 0.004) with a higher number of cases and higher proportion of positive cases during the dry season (n = 173; 74.3%) when compared to the wet season (n = 111; 61.0%) (Table 3). There was a trend in the increase of monthly rabies cases from the start of the dry season (November) through to the start of the wet season (May) cases over the sampling period (Fig 4). Despite no significant monthly trend in rabies positive cases from January to December (Chi^2^ = 1.38; p = 0.240) there was a significant decrease in rabies cases in the 12-month period May to through to April (Chi^2^ = 8.87; p = 0.003) (Fig 4).

### Host factors

There was no significant difference in the proportion of rabies positive cases between the sexes (males 62.3%; Chi^2^ = 2.12; p = 0.145) or age groups (Chi^2^ = 4.32; p = 0.363) (Table 3). There was no significant trend in rabies cases with increasing age (Chi^2^ = 2.17; p = 0.141). The median age of rabies-positive animals (12 months; interquartile range (IQR) 4–24) was, however, more than double the median age of the negative animals (5 months (IQR: 3–12 months).

## Discussion

The results presented here clearly demonstrate that dog rabies remains a significant public health problem in Lao PDR. During the period from 2010 through 2016, the number of dog rabies cases were reasonably stable, ranging from 29 to 57 cases annually. Previous studies [4, 13, 14] have reported somewhat higher annual numbers of dog rabies cases in Lao PDR between 1988 and 2009 and there was an apparent increase in the number of rabies cases during 1992 and 1996 (Table 1) although the proportion of rabies-positive cases were similar to those reported here. In the period from 2004–2011 there were 635 laboratory-confirmed animal rabies cases (51.1% of submissions) [4] in Lao PDR, and Kamsing et al. [15] (cited by [16]) stated that from 1993 to April 2008, a total of 2813 dog brain samples from central and southern Lao PDR were examined, of which 1308 (46.5%) were rabies positive. There is no clear reason why the number of samples submitted for diagnosis should have decreased during the period of the study and it is interesting to note that the proportion of rabies-positive cases was similar or slightly higher than those previously reported. However, historical results, especially those based on tests conducted in Lao PDR prior to 2000, should be treated with caution due to lower accuracy of the Sellers stain microscopy to detect Negri bodies in comparison with the more sensitive DFAT [17, 18]. The numbers and provincial distribution of rabies cases presented here are similar to those reported by Ahmed et al. [4], who examined the epidemiology of rabies in Lao PDR from 2004–2011 and also found that the majority of cases were located in Vientiane Capital, Champassak and Vientiane Province. However, it is likely this observation may be influenced by sampling bias due the closer proximity of these provinces to diagnostic facilities and knowledge regarding rabies control and dog-bite treatment.

This study has demonstrated a significantly higher proportion of rabies-confirmed cases during the dry season (<100 mm monthly rainfall) compared to the wet season. A possible explanation for this difference may be that animals are less likely to socialize during periods of high rainfall leading to a reduction in rabies transmission. Seasonality in dog rabies has previously been reported in Peru [19], Chile [20], Bolivia [21] and the United States [22], where the springtime excess of cases has been associated with greater contact between animals during the mating season, factors relating to herd immunity and an increased number of susceptible animals [19].

Male dogs accounted for a higher proportion of rabies-positive animals than females, although the association was not significant. This observation has been made previously in Florida in the USA [23], Nigeria [24] and Ghana [25] and is thought to be due to an association with the breeding season when it was observed that male dogs were fighting each other for the same bitch resulting in wounds that may have led to rabies infections.

This study has a number of limitations. A major constraint in determining the true burden of animal rabies in Lao PDR is the passive nature of surveillance. At present, the only source of samples for rabies diagnosis are those submitted following an animal bite incident, of which the majority are caused by dogs. This is likely to greatly underestimate the true number of rabies cases and may only represent the “tip of the iceberg”. Furthermore there is little information on the human:dog ratio for comparison of rabies risks. Another limitation is the fact that surveillance was confined to the increasingly urbanized Vientiane Capital, where reference diagnostics such as the DFAT are available, albeit only at NAHL, which severely limits the capacity to estimate the true number of rabies cases throughout the Lao PDR as there is comparatively limited capacity for rabies diagnosis outside of Vientiane Capital. Interprovincial submission of specimens for rabies diagnosis rarely occurs due to financial constraints or lack of awareness of the need to submit samples for reference diagnosis, further compounding the underreporting of both animal and human rabies. Another limitation of the study is that it has exclusively focused on canine rabies however the occurrence of rabies and other lyssavirus infections in wildlife, livestock and other domestic animals is a distinct possibility and the lack of samples from other species is likely to be due to the restricted capacity of the diagnostic service and the low level of awareness of rabies infections in these species. In this study, majority of samples came from dogs with suspected rabies infection, as dog bite was the primary reason for presentation at NAHL.

The availability of simple, accurate and affordable alternatives to the DFAT, such as rabies antigen rapid diagnostic tests (RDTs) [26–29], would be a useful adjunct to current surveillance methods in rural Lao PDR. However, a recent study has highlighted diagnostic problems with these tests [30] and therefore rabies antigen RDTs results should be interpreted with caution and may only be useful for surveillance purposes rather than for acute diagnosis and patient management (i.e., post-exposure prophylaxis).

Successful control and eradication of rabies is best achieved by an effective and targeted dog vaccination programs. Achieving acceptable levels of herd immunity to control rabies in the dog population is dependent on effective and consistent vaccination rates. Regarding rabies-control in Lao PDR, the government has an active program with the aim of rabies elimination, including advocacy by the authorities for the prevention and control of rabies, mobilization of community involvement, dog vaccination, allocation of resources, promotion of public awareness, provision of post-exposure prophylaxis and facilitation of the coordination between human and animal health sectors [5]. Rabies vaccination is largely focused on the major population centers of Lao PDR encouraging dog owners to vaccinate their pets which has led to the emergence of local veterinary practitioners in small animal clinics who provide vaccination services. Unfortunately, there is limited and unreliable data on the types of vaccines that are being administered. International partners have supported vaccination programs in Lao PDR in the recent past, with 50,000 doses of vaccine from the OIE Rabies Regional Vaccine Bank funded by the European Union in September 2012 followed by a further delivery of 120,000 additional doses [5] Such vaccinations are normally administered during mass vaccination campaigns such as World Rabies Day (28 September) or World Immunization Week and comprise a substantial proportion of the vaccines recorded as administered during these campaigns. However, maintaining the momentum of such programs in resource limited settings without continued external resources remains an issue. The challenge remains to find effective means to secure effective and safe vaccines via national and international disease control programs and then apply them in a strategic manner to achieve consistent and effective rabies control.

Other factors to control rabies in Lao PDR are also required. Stray dogs remain a problem throughout rural and urban areas of Lao PDR and may significantly contribute to the spread of rabies however the influence of stray dogs on rabies epidemiology in Lao PDR is not well understood and requires further investigation. Transboundary movement of dogs has the potential to impact the epidemiology of rabies in Lao PDR given that the country shares borders with China, Thailand, Cambodia and Vietnam. However, the official transboundary movement of dogs is strictly regulated with the transit of dogs for trade purpose prohibited and pets imported and exported must be rabies vaccinated. Wildlife and domesticated non-canine species are also potential reservoirs and vectors for rabies however their influence on rabies spread in Lao PDR is poorly understood and further studies are required to understand the influence of these animals on rabies epidemiology in Lao PDR.

### Conclusion

Dog bite remains the main source of rabies transmission in the Lao PDR and dog vaccination remains the best method for rabies control. Dog owners can play an important role in rabies-control activities by ensuring that their dogs are vaccinated and restrained to reduce the opportunity for bites by rabies-infected animals. Governments and donors have an equally important role to play in providing a conducive environment and by facilitating increased awareness through mass media campaigns, effective delivery of vaccination and surveillance so that the situation can be actively monitored nationwide.
